# Identification of SARS-CoV-2 Main Protease Inhibitors from a Library of Minor Cannabinoids by Biochemical Inhibition Assay and Surface Plasmon Resonance Characterized Binding Affinity

**DOI:** 10.3390/molecules27186127

**Published:** 2022-09-19

**Authors:** Chang Liu, Tess Puopolo, Huifang Li, Ang Cai, Navindra P. Seeram, Hang Ma

**Affiliations:** 1 Department of Biomedical and Pharmaceutical Sciences, College of Pharmacy, University of Rhode Island, Kingston, RI 02881, USA; 2 Cannabis Research Collaborative, College of Pharmacy, University of Rhode Island, Kingston, RI 02881, USA

**Keywords:** SARS-CoV-2, COVID-19, main protease (M^pro^), minor cannabinoids, decarboxylation, structure and activity relationship, surface plasmon resonance, binding

## Abstract

The replication of the severe acute respiratory syndrome coronavirus 2 (SARS-CoV-2) is mediated by its main protease (M^pro^), which is a plausible therapeutic target for coronavirus disease 2019 (COVID-19). Although numerous in silico studies reported the potential inhibitory effects of natural products including cannabis and cannabinoids on SARS-CoV-2 M^pro^, their anti-M^pro^ activities are not well validated by biological experimental data. Herein, a library of minor cannabinoids belonging to several chemotypes including tetrahydrocannabinols, cannabidiols, cannabigerols, cannabichromenes, cannabinodiols, cannabicyclols, cannabinols, and cannabitriols was evaluated for their anti-M^pro^ activity using a biochemical assay. Additionally, the binding affinities and molecular interactions between the active cannabinoids and the M^pro^ protein were studied by a biophysical technique (surface plasmon resonance; SPR) and molecular docking, respectively. Cannabinoids tetrahydrocannabutol and cannabigerolic acid were the most active M^pro^ inhibitors (IC_50_ = 3.62 and 14.40 μM, respectively) and cannabigerolic acid had a binding affinity $K_{D}=2.16\times 10^{-4}$ M). A preliminary structure and activity relationship study revealed that the anti-$M^{\mathrm{pro}}$ effects of cannabinoids were influenced by the decarboxylation of cannabinoids and the length of cannabinoids’ alkyl side chain. Findings from the biochemical, biophysical, and computational assays support the growing evidence of cannabinoids’ inhibitory effects on SARS-CoV-2 M^pro^.

## 1. Introduction

Severe acute respiratory syndrome coronavirus 2 (SARS-CoV-2) is the etiological cause of the coronavirus disease 2019 (COVID-19), which led to a global pandemic due to its high infection rate and lack of effective treatments to cure this disease [1,2]. To combat this unprecedented health crisis, the scientific community is rallying to develop potential treatments for COVID-19 [3]. Among these efforts, in addition to the vaccine, small molecules including natural products may play an immense role in the management of this devastating disease. Natural products have been extensively studied for antiviral effects [4,5] and some are considered as potential interventions for coronavirus-related infections [6]. The screening of natural products with anti-coronavirus effects is often conducted in enzyme-based biochemical assays [7], cell-based methods [8], and in vivo models [9]. Additionally, in silico approaches such as virtual screening of natural product-based antivirals may offer valuable insights for exploring natural products’ potential as therapeutic agents against COVID-19 [10].

Recent pre-clinical studies support that natural products may suppress the growth of coronavirus via the inhibition of viral RNA replication-related enzymes [6,11]. Among these enzymes, the SARS main protease (M^pro^; also known as 3-chymotrypsin-like protease, 3CL^pro^) is a key enzyme responsible for mediating virus replication and transcription by cleaving two replication related polyproteins, namely, polyprotein 1a and polyprotein 1ab [12]. The homology model of SARS-CoV-2 M^pro^ is known, and its X-ray crystal structure became available soon after the outbreak of COVID-19, which provides a structural basis for the discovery of SARS-CoV-2 inhibitors using in silico approaches [12,13]. The crystal structure of SARS-CoV-2 M^pro^ that was co-crystallized with the N3 inhibitor was reported in 2020, which facilitated the discovery of M^pro^ inhibitors. SARS-CoV-2 M^pro^ is composed of three domains including domain I (residues 8-101), domain II (residues 102-184), and domain III (residues 201-303) [14,15]. Domains I and II are highly conserved, whereas domain III has more variable regions on the helical area and surface loops. The substrate-binding site of SARS-CoV-2 M^pro^ is located in a cleft between domains I and II, which is the targeting area for several known M^pro^ inhibitors, including N1 and N3 [14,15].

SARS-CoV-2 M^pro^ is considered as a viable target for the development of therapeutic agents for SARS-CoV-2-related infections. Numerous natural products including extracts of medicinal plants and their phytochemicals have been reported to show inhibitory effects on SARS-CoV-2 M^pro^. Due to the limitation of feasible in vitro assays that were available at the time of the outbreak of COVID-19, a number of studies utilized in silico molecular docking methods to identify natural products with the potential binding capacity to the M^pro^ protein [16,17,18,19]. The recent development of antiviral bioassays using cell-free and cell-based models facilitated the screening of natural products with anti-M^pro^ activity. For instance, several natural products including flavonoids showed inhibitory effects on SARS-CoV-2 M^pro^ (in a biochemical assay) and suppressive effects on virus replication (in a cell-based assay) [18]. In addition, our group has developed a method to evaluate the anti-M^pro^ activity of natural products using a combination of an enzymatic assay, a biophysical tool, namely, surface plasmon resonance (SPR), and a computational assay (docking) [19]. Based on these combinatorial assays, several natural products including ellagitannins and gallotannins were identified as promising SARS-CoV-2 M^pro^ inhibitors.

Notably, published pre-clinical studies support bioactive compounds including cannabinoids from the *Cannabis* species may exert promising antiviral effects [20,21]. For instance, virtual screening has identified five hits with strong binding energies with substrate-binding site of SARS-CoV-2 M^pro^ from a panel of 32 cannabinoids [22]. Further in vitro studies have revealed that Δ^9^-tetrahydrocannabinol (Δ^9^-THC) and cannabinol are potent inhibitors against SARS-CoV-2 M^pro^ with IC_50_ values of 10.25 and 7.91 μM, respectively [22]. Additionally, an in silico study reported that cannabinoids including cannabichromanon, cannabinolic acid, and cannabinol had a free binding ΔG of −33.63, −23.24, and −21.60 kcal/mol, respectively, at the active site of SARS-CoV-2 M^pro^ [23]. However, to date, it remains unclear whether cannabinoids including the major ones (e.g., cannabidiol; CBD) and other minor ones can confer antiviral effects via the inhibition of SARS-CoV-2 M^pro^. Our group initiated a program to evaluate the biological effects of a library of cananbinoids, which revealed that cannabinoids, such as CBD and several minor cannabinoids, confer inhibitory effects against the activities of a panel enzymes including α-glucosidase [24], caspase-1 [25], and acetylcholinesterases (A and B) [26]. As continued research efforts, herein, we used a biochemical based enzymatic assay to evaluate the anti-M^pro^ activity of a series of cannabinoids including CBD and a collection of minor cannabinoids. These minor cannabinoids are in various chemotypes including tetrahydrocannabinols (THCs), cannabidiols (CBDs), cannabigerols (CBGs), cannabichromenes (CBCs), cannabinols (CBNs), cannabicyclols (CBLs), cannabitriols (CBTs), and miscellaneous cannabinoids. In addition, the binding affinities between cannabinoids and the M^pro^ protein were characterized by a biophysical technique (i.e., the SPR assay). Furthermore, we used computational docking experiments to depict the molecular interactions between cannabinoids and the M^pro^ protein. Based on data from the biochemical, biophysical, and computational experiments, a preliminary structure–activity relationship (SAR) of cannabinoids’ inhibitory effects on SARS-CoV-2 M^pro^ was also explored.

## 2. Results

The minor cannabinoids included in the anti-M^pro^ assay were: THCB (Δ^9^-tetrahydrocannabibutol), 11-OH-THC 11-nor-Δ^8^-tetrahydrocannabinol-9-Carboxylic acid), THCP (Δ^9^-tetrahydrocannabiphorol), 11-nor-9-carboxy-THC (11-nor-9-carboxy-Δ^8^-tetrahydrocannabinol), Δ^8^-THCA-A (Δ^8^-trans-tetrahydrocannabinolic acid A),THCV (Δ^9^-tetrahydrocannabivarinic acid A), THCA-A (Δ^9^-tetrahydrocannabinolic acid A), CBDA (cannabidiolic acid), CBDB (cannabidibutol), 6α-OH-CBD (6α-hydroxy cannabidiol), CBDAME (cannabidiolic acid methyl ester), CBD (cannabidiol), CBDP (cannabidiphorol), CBDV (cannabidivarin), CBGA (cannabigerolic acid), CBG (cannabigerol), CBGV (cannabigerovarin), CBGO (cannabigerorcin), CBGM (cannabigerol monomethyl ether), CBGOA (cannabigerorcinic acid), CBC (cannabichromene), CBCVA (cannabichromevarinic acid), CBCA (cannabichromenic acid), CBL (cannabicyclol), CBN (cannabinol), CBND (cannabinodiol), CBT (cannabicitran).

### 2.1. Inhibitory Effects of the THC-Type Cannabinoids on SARS-CoV-2 M^pro^

**Figure 1:**
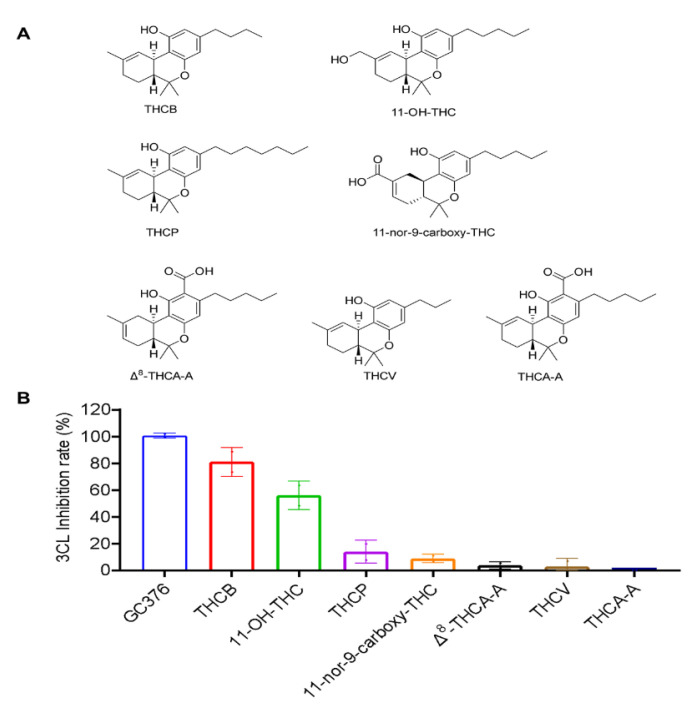
The chemical structures of the THC-type of cannabinoids (**A**) and their inhibitory effect on the activity of SARS-CoV-2 M^pro^ (**B**). These cannabinoids were tested at a concentration of 10 μM with the MBP-tagged SARS-CoV-2 M^pro^ inhibition assay. GC376 was used as the positive control and each test compound was assayed in triplicate.

We first evaluated the inhibitory effects of a group of THC-type cannabinoids (10 μM; Figure 1A), including THCB,11-OH-THC, THCP, 11-nor-9-carboxy-THC, Δ^8^-THCA-A, THCV and THCA-A, on the activity of SARS-CoV-2 M^pro^. THCB and 11-OH-THC showed the most active anti-M^pro^ effect with an inhibition rate of 81.0% and 56.0%, respectively. Other cannabinoids in this group only showed weak inhibitory effects on SARS-CoV-2 M^pro^ within a range of inhibition from 2.6% to 13.8%.

### 2.2. Inhibitory Effects of the CBG-Type Cannabinoids on SARS-CoV-2 M^pro^

**Figure 2:**
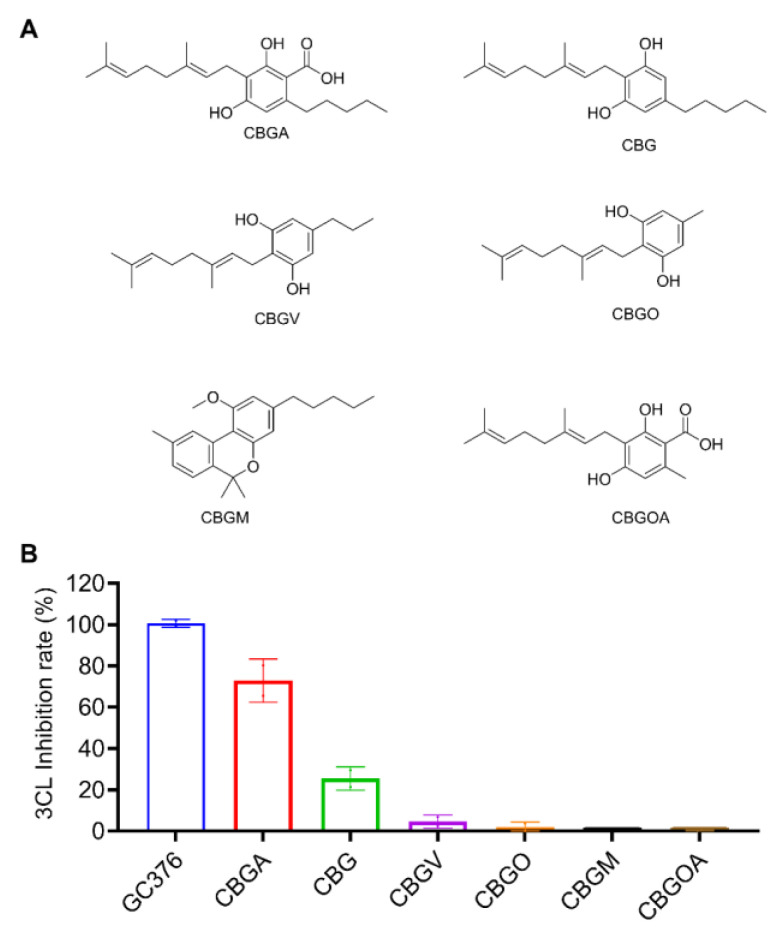
The chemical structures of the CBG-type of cannabinoids (**A**) and their inhibitory effect on the activity of SARS-CoV-2 M^pro^ (**B**). These cannabinoids were tested at a concentration of 10 μM with the MBP-tagged SARS-CoV-2 M^pro^ inhibition assay. GC376 was used as the positive control and each test compound was assayed in triplicate.

Next, CBGA was identified as a SARS-CoV-2 M^pro^ inhibitor from the CBG-type cannabinoids (Figure 2A). At a concentration of 10 μM, CBGA inhibited the activity of SARS-CoV-2 M^pro^ by 72.87%. The decarboxylated form of CBGA, namely, CBG, showed a moderate anti-M^pro^ activity with an inhibition rate of 25.44%. Other CBG-type cannabinoids were inactive (inhibitory rate < 10%; Figure 2B).

### 2.3. Inhibitory Effects of the CBD-Type Cannabinoids on SARS-CoV-2 M^pro^

**Figure 3:**
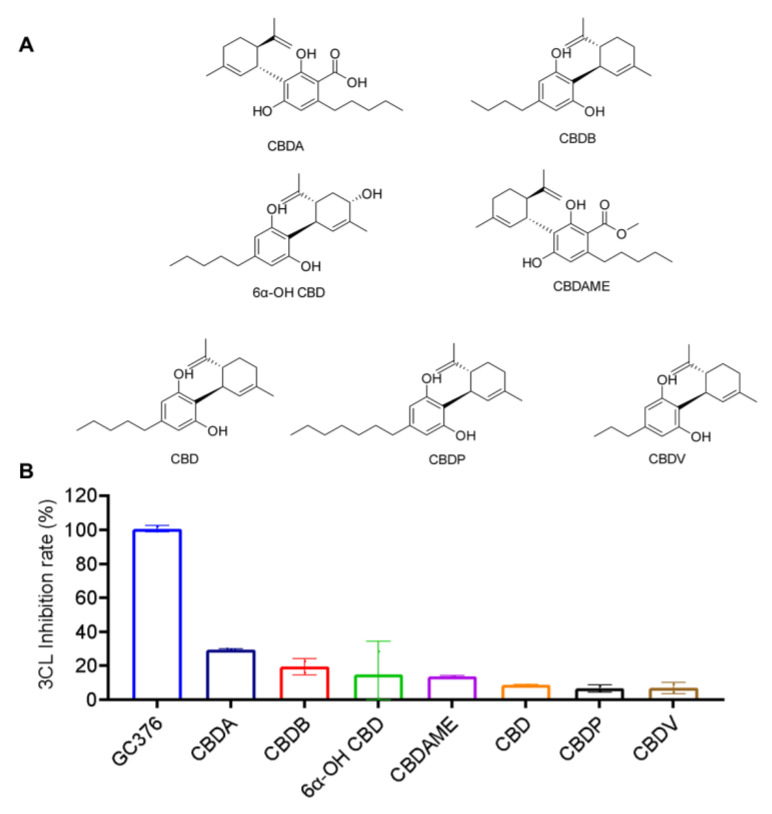
The chemical structures of the CBD-type of cannabinoids (**A**) and their inhibitory effect on the activity of SARS-CoV-2 M^pro^ (**B**). These cannabinoids were tested at a concentration of 10 μM with the MBP-tagged SARS-CoV-2 M^pro^ inhibition assay. GC376 was used as the positive control and each test compound was assayed in triplicates.

The anti-M^pro^ activity of the CBD-type cannabinoids (10 μM) including CBDA, CBDB, 6α-OH CBD, CBDAME, CBD, CBDP, and CBDV (chemical structures shown in Figure 3A) was evaluated. In general, compounds in this group showed weak inhibitory effects on SARS-CoV-2 M^pro^. CBDA, CBDB, 6A-OH CBD, CBDAME, CBD, CBDP and CBDV inhibited the M^pro^ activity by 29.4%, 19.38%, 14.68%, 13.71%, 8.74%, 6.55% and 6.87%, respectively.

### 2.4. Inhibitory Effects of CBC-Type Cannabinoids on SARS-CoV-2 M^pro^

**Figure 4:**
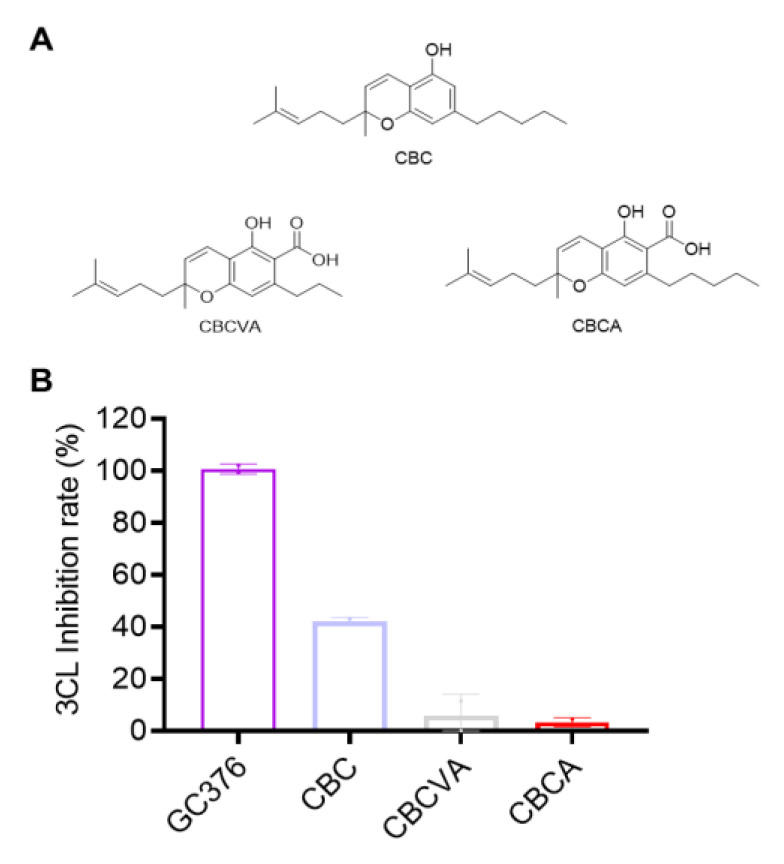
The chemical structures of the CBC-type of cannabinoids (**A**) and their inhibitory effect on the activity of SARS-CoV-2 M^pro^ (**B**). These cannabinoids were tested at a concentration of 10 μM with the MBP-tagged SARS-CoV-2 M^pro^ inhibition assay. GC376 was used as the positive control and each test compound was assayed in triplicate.

Three CBC-type compounds including CBC, CBCVA and CBCA were tested for their anti-M^pro^ activity at 10 μM (chemical structures shown in Figure 4A). Only CBC showed a moderate inhibitory effect on SARS-CoV-2 M^pro^ (42.13% inhibition). CBCVA and CBCA were not active (inhibition rate < 10%; Figure 4B).

### 2.5. Inhibitory Effects of Other Cannabinoids on SARS-CoV-2 M^pro^

**Figure 5:**
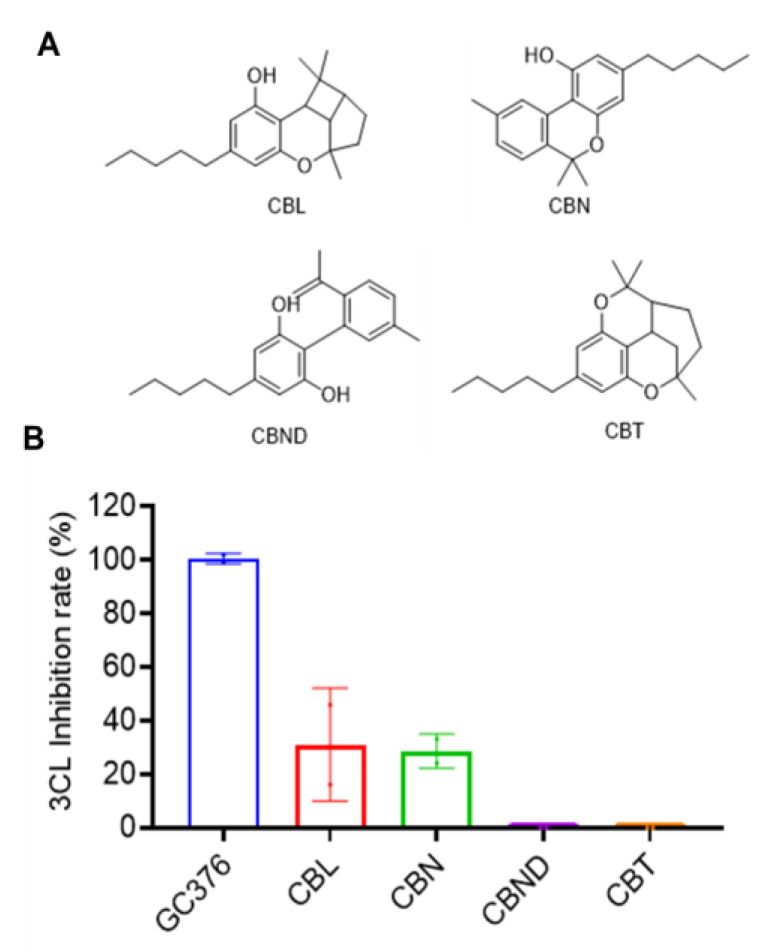
The chemical structures of other types of cannabinoids (**A**) and their inhibitory effect on the activity of SARS-CoV-2 M^pro^ (**B**). These cannabinoids were tested at a concentration of 10 μM with the MBP-tagged SARS-CoV-2 M^pro^ inhibition assay. GC376 was used as the positive control and each test compound was assayed in triplicate.

Four compounds including CBL, CBN, CBND and CBT were tested for their anti-M^pro^ activity at 10 μM (chemical structures shown in Figure 5A). CBL and CBN showed a weak inhibitory effect on SARS-CoV-2 M^pro^ (31.11% and 28.74% inhibition, respectively), whilst CBND and CBT were not active (inhibition rate < 10%; Figure 5B).

### 2.6. Validation of the SPR Method to Measure the Binding Affinity with SARS-CoV-2 M^pro^ Protein

**Figure 6:**
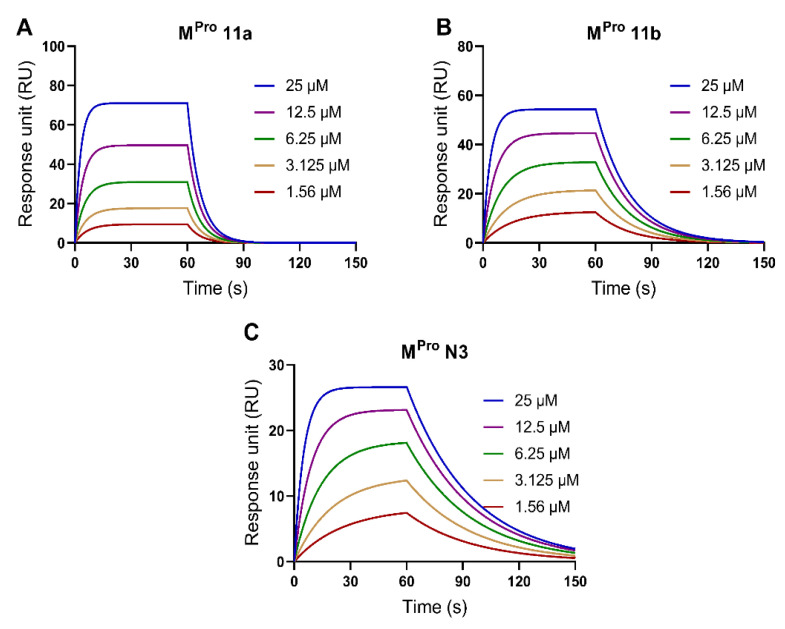
Validation of the SPR method to measure the binding affinities between the M^pro^ protein and several known SARS-CoV-2 M^pro^ inhibitors including M^pro^ 11a (**A**), M^pro^ 11b (**B**), and M^pro^ N3 (**C**).

We used a label-free biosensor-based SPR assay to study the binding affinities between cannabinoids and SARS-CoV-2 M^pro^ protein. First, the experimental conditions of the SPR model were optimized with the positive controls including compounds M^pro^ 11a, M^pro^ 11b, and M^pro^ N3, which all showed potent anti-M^pro^ activity (inhibition > 98% at 10 μM; Supplementary Materials Figure S1). The M^pro^ protein was properly coated on the SPR sensor chip. Next, the SPR assays depicted the binding profiles of the inhibitors M^pro^ 11a, M^pro^ 11b (both at 1.56–25 μM), and M^pro^ N3 (at 1.56–50 μM) with a RU ranging from 9.5–71.1, 12.4–54.3 and 7.4–26.6, respectively, in the SPR sensorgrams (Figure 6). The binding parameters including association constant (*K*_a_), dissociation constant (*K*_d_), and affinity values (*K*_D_) of M^pro^ 11a, M^pro^ 11b, and M^pro^ N3 were determined from the SPR data (Table 1).

### 2.7. Decarboxylation of CBGA and CBDA Attenuates the Anti-M^pro^ Activity

**Figure 7:**
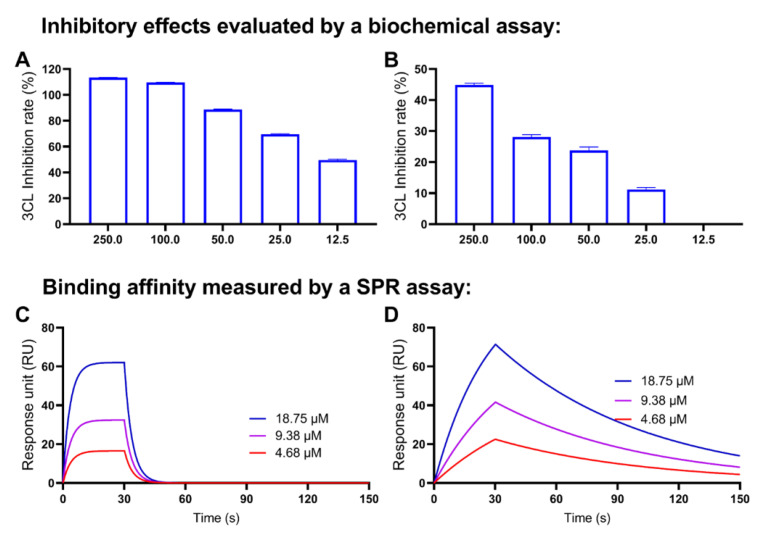
Concentration-dependent inhibitory effects of the CBG-type cannabinoids on the M^pro^ activity (evaluated by a biochemical enzyme inhibition assay) and their binding capacity to the M^pro^ protein (measured by the SPR assay). The IC_50_ values of CBGA (**A**) and CBG (**B**) were calculated by data from a biochemical assay with the GraphPad Prism8. The binding affinities between CBDA or CBD and the M^pro^ protein were determined by the SPR assay with a representative SPR sensorgram of the binding between CBGA (**C**) or CBG (**D**) and the M^pro^ protein.

Several cannabinoids with promising activity in the anti-M^pro^ screening assay were further evaluated for their binding capacity with the M^pro^ protein using the SPR assay. In addition, a preliminary structure and activity relationship (SAR) for selected cannabinoids and their anti-M^pro^ activity was explored. First, the inhibitory effects of a cannabinoid acid (i.e., CBGA) and its decarboxylated form (CBG) were compared. CBGA showed superior anti-M^pro^ activities to CBG at the concentration at 250, 100, 50, 25, 12.5 μM (inhibition rate of 72.9%, 18.2%, 18.5%, 8.0% and 6.0%, respectively; Figure 7A,B). CBGA had a lower IC_50_ as compared to CBG (20.7 vs. >250 μM, respectively).

Furthermore, the binding affinity of CBGA and CBG was compared by the SPR assay. As shown in the SPR sensorgrams, CBGA displayed a typical binding association–dissociation pattern with RU values ranging from 16.6–62.1 (Figure 7C), whilst CBG had a fast dissociation phase (Figure 7D). The M^pro^ protein binding capacity of CBGA and CBG was supported by their binding parameters, in which only CBGA had detectable *K*a, *K*d, and *K*_D_ values (Table 2).

**Figure 8:**
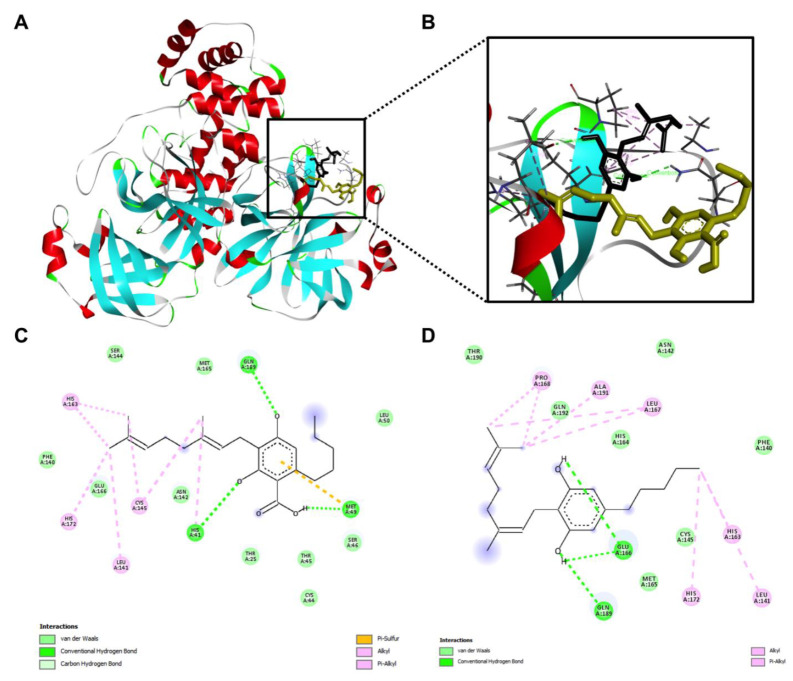
The overall view of the computational docking depicted interactions between the M^pro^ protein and CBGA and CBG (**A**) and the zoom-in view of the ligand-protein interaction with CBGA (shown in golden) and CBG (shown in black) stacked with each other (**B**). Molecular forces formed between CBGA (**C**) or CBG (**D**) and the M^pro^ protein.

In addition, the interactions between the M^pro^ protein and CBGA and CBG were explored. Computational docking showed that both CBGA and CBG can fit into the binding pocket of the M^pro^ protein (Figure 8A,B) but they formed distinct molecular bonds with the protein. Although the major molecular force in the ligand–protein complex was conventional hydrogen bonding, CBGA was able to form hydrogen bonds via different amino acid residues, due to the presence of the carboxylic acid, as compared to CBG (His 41, Met 49 and Glu 166 vs. Glu 166 and Arg 189, respectively; Figure 8C,D).

The estimated binding energies, including the free energy of binding, intermolecular energy, total internal energy, and torsional free energy, suggested that CBG’s binding energies were lower than CBGA’s (Table 3).

**Figure 9:**
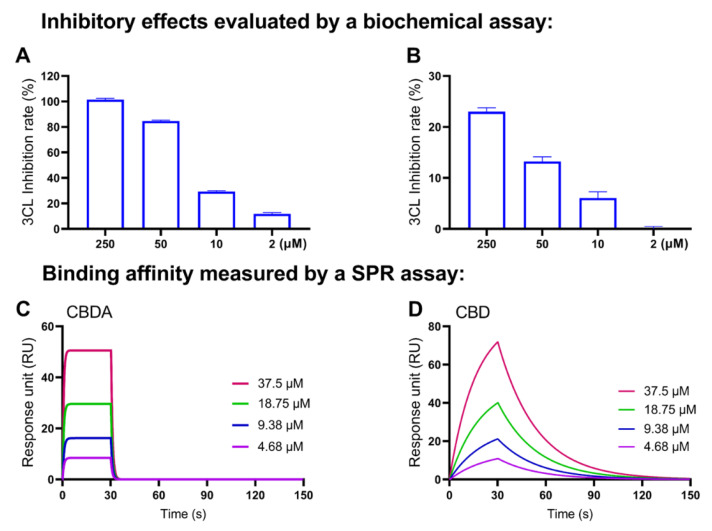
Concentration-dependent inhibitory effects of the CBD-type cannabinoids on the M^pro^ activity (evaluated by a biochemical enzyme inhibition assay) and their binding capacity to the M^pro^ protein (measured by the SPR assay). The IC_50_ values of CBDA (**A**) and CBD (**B**) were calculated by data from a biochemical assay with the GraphPad Prism8. The binding affinities between CBDA or CBD and the M^pro^ protein were determined by the SPR assay with a representative SPR sensorgram of the binding between CBdA (**C**) or CBD (**D**) and the M^pro^ protein.

A similar SAR pattern, in which the decarboxylation decreased the anti-M^pro^ activity of cannabinoids, was observed in another pair of cannabinoids: CBDA and CBD. CBDA inhibited the M^pro^ activity by 101.43%, 84.75%, 29.40% and 11.88% at concentrations of 250, 50, 10 and 2 μM, respectively (Figure 9A). CBD only showed weak inhibitory effects on SARS-CoV-2 M^pro^ at higher concentrations (250, 50 and 10 μM) by 23.03%, 13.23% and 6.10%, respectively. The SPR assay detected the binding capacity of CBDA and CBD with RUs ranging from 4.3–50.6 and 10.9–71.0 (Figure 9C,D) and their binding parameters (Table 4).

**Figure 10:**
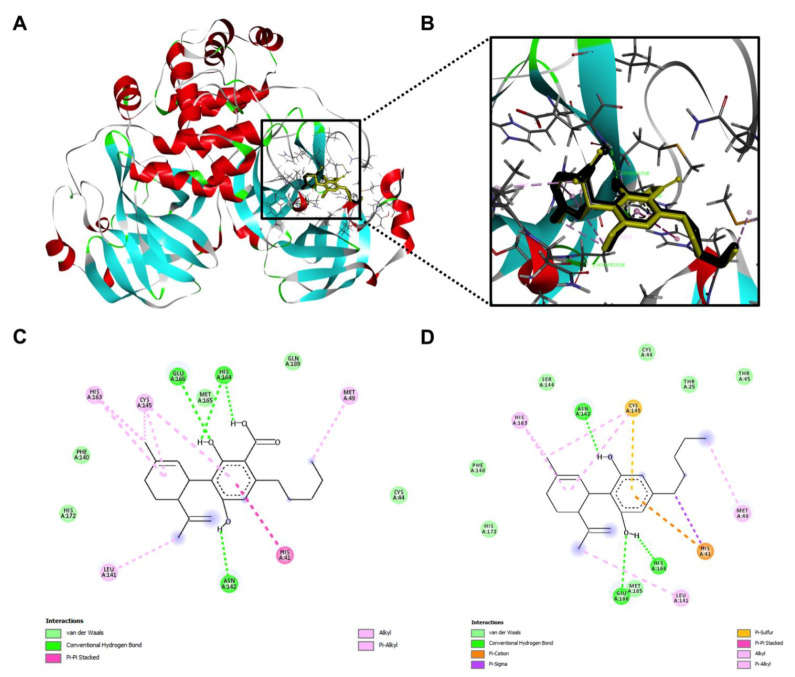
The overall view of the computational docking depicted interactions between the M^pro^ protein and CBDA and CBD (**A**) and the zoom-in view of the ligand-protein interaction with CBDA (shown in golden) and CBD (shown in black) stacked with each other (**B**). Molecular forces formed between CBDA (**C**) or CBD (**D**) and the M^pro^ protein.

Data from the molecular docking experiments illustrated the potential impact of the decarboxylation of CBDA on the ligand–protein interactions (Figure 10). CBDA and CBD both have several common molecular forces (such as van der Waals, hydrogen bond, alkyl, and Pi alkyl) that contributed to the ligand-protein binding. CBDA and CBD formed hydrogen bonds at the same locations on chain A (e.g., Asn 142 and His 164, and Glu 166, respectively; Figure 10C,D). The molecular docking study predicted that this pair of compounds had a similar binding capacity whilst CBD had lower binding energies than CBDA (Table 5).

## 3. Discussion

The antiviral effects of cannabis and cannabinoids against SARS-CoV-2, along with their mechanisms of action, have been studied. Two carboxylated cannabinoids, namely, CBGA and CBDA, were reported to block the infection of a pseudovirus expressing the SARS-CoV-2 spike protein in a cellular model with human epithelial cells, suggesting that cannabinoids may prevent the SARS-CoV-2 virus from entering cells [27]. Cannabis extracts and the major cannabinoid, CBD, may also confer preventive effects against SARS-CoV-2 by modulating the expression of the angiotensin-converting enzyme 2 (ACE2), which is a pathway for the virus entering human cells, as shown in artificial 3D human models of oral, airway and intestinal tissues [28]. Apart from the virus spike protein and ACE2, the 3CL M^pro^ is also a critical molecular target for the prevention of the replication of SARS-CoV-2. The anti-M^pro^ potential of a handful of cannabinoids including Δ^9^-THCA, Δ^9^-THC, CBN, CBD and CBDA was studied using in silico methods (by molecular dynamic simulation and docking) [22]. However, the understanding of minor cannabinoids’ effects on the SARS-CoV-2 3CL M^pro^ is limited by some shortcomings, such as a lack of experimental evidence and a confined selection of minor cannabinoids. Data from our current study provided experimental evidence to support previously reported inhibitory effects of cannabinoids against the SARS-CoV-2 3CL M^pro^. These compounds (at 10 μM) showed a wide range of inhibition capacity from promising (e.g., TCHB and CBGA; inhibition rate > 70%) to moderate (inhibition rate < 40%) effects, along with the majority of cannabinoids showed insignificant inhibitory effects (inhibition < 5%). Varied anti-M^pro^ effects observed in the enzyme inhibition assay suggest that the biological activity of cannabinoids may be influenced by their chemical structures. This was confirmed by their binding affinity to the M^pro^ protein obtained from the SPR binding assay, where cannabinoids with different chemical moieties (e.g., carboxylic acid) showed distinct binding capacities (Figure 7 and Figure 9).

**Figure 11:**
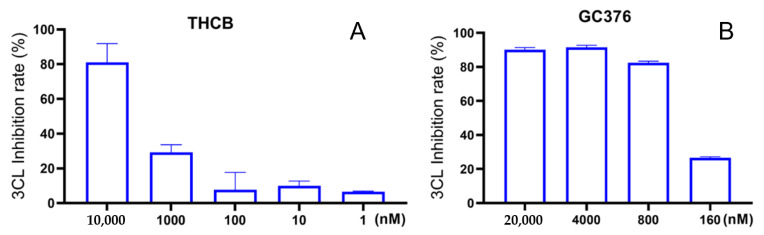
The concentration-dependent anti-M^pro^ activity of THCB (**A**) and the positive control GC376 (**B**) measured by a biochemical assay.

It should be noted that several discrepancies were observed in this study. First, although data from the SPR assay showed that cannabinoids with strong anti-M^pro^ activity (for instance, CBGA) were able to bind to the M^pro^ protein, an exception was observed where the binding affinity of the most active cannabinoid, namely, THCB (IC_50_ = 3.62 μM), was not able to be measured in the SPR assay. This is similar to one of the positive controls, GC376, which also showed a potent enzyme inhibitory effect (IC_50_ = 0.61 μM) but no binding was detected in the SPR assay, whereas other positive controls’ binding profiles were measured using the same SPR binding protocol (Figure 11).

Several factors may have contributed to this observation. For example, it is possible that THCB and GC376 had a different binding pocket (as compared to the other positive controls) on the M^pro^ protein, where the surface of the binding pocket may not be displayed on the SPR sensor chip properly. This requires further method development for the SPR assay with different instrumental conditions (e.g., types of sensor chips and protein coating approach). Alternatively, other biophysical tools, such as isothermal titration calorimetry and saturation transfer differentiation-based nuclear magnetic resonance, may be applied to confirm the binding affinity between the active cannabinoids and the M^pro^ protein. Second, a discrepancy was observed in the inhibitory effects of cannabinoids obtained from the biochemical assay and from the in-silico predictions. Data from the biochemical assay showed that the decarboxylated cannabinoids, e.g., CBG and CBD, had weaker inhibitory effects against the SARS-CoV-2 3CL M^pro^ as compared to their respective carboxylated precursors, CBGA and CBDA (Figure 7 and Figure 9). However, this was in contradiction to their computational predicted inhibition constant, where CBG was more active than CBGA, as well as CBD was more active and CBDA. Additionally, the docking-based ligand–protein interactions from our current study were compared with previously reported data, in which two pairs of cannabinoids (CBD-CBDA and CBG-CBGA) had similar binding energies [23]. The contradictions in data from the docking studies and biochemical assays suggest that, although it is common to use computational docking to explore the inhibitory effects of natural products (i.e., minor cannabinoids in this study), it is critical to use experimental assays to validate results from the computational assays to avoid false positive results. Third, apart from the impact of decarboxylation on cannabinoids, only a preliminary SAR pattern was observed in the current study, where the length of the alkyl sidechain on the structures of the THC-type and CBD-type cannabinoids may influence their anti-M^pro^ activity. For instance, THCB and CBDB (also known as THC-C4 and CBD-C4, respectively) showed stronger anti-M^pro^ activity, whilst THCV (also known as THC-C2) and CBDV (CBD-C2) had weaker anti-M^pro^ activity, as compared to THC and CBD, respectively. However, it is not clear (1) the most favorable length of the alkyl side chain for cannabinoids’ inhibitory effects on the SARS-CoV-2 M^pro^, and (2) whether this SAR pattern can be applied to other chemotypes of cannabinoids. Several factors should be considered for addressing these issues. Although a commercially available anti-M^pro^ assay and a docking model were used in the current study, the parameters of these assays should be optimized for further evaluations of the impact of the length of the alkyl side chain of cannabinoids on their anti-M^pro^ activities. For example, the dynamic changes in the enzyme-inhibition assay should be monitored after adding the substrate solution for a better understanding of the test compounds’ inhibitory patterns. In addition, further studies including the chemical synthesis and biological evaluations of cannabinoids derivatives are warranted to better elucidate the SAR of cannabinoids on the SARS-CoV-2 M^pro^ enzyme. The impact of subgroups of different chemotypes of cannabinoids and their chemical features on the anti-M^pro^ activity should be further examined. For instance, the THC-type of cannabinoids (with one additional cyclized oxygen-containing ring as compared to the two 6-carbon rings for the CBD-type compounds) seemed to be more active than the CBD analogs. This may be attributed to the stronger molecular interactions between the THC-type cannabinoids and the M^pro^ protein. Moreover, the presence of specific chemical moieties, such as the prenyl or geranyl groups, may have contributed to the anti-M^pro^ activity of the CBG-type of cannabinoids. This is not surprising given that reported studies support that these functional groups may lead to enhanced biological activity [29,30]. However, limited access to structurally diverse natural cannabinoids can be a barrier to the full elucidation of their SAR on the SARS-CoV-2 M^pro^. Future investigations including the synthesis of cannabinoid analogs for further anti-M^pro^ evaluations are warranted. Nevertheless, our findings from biochemical (enzyme inhibition assay) and biophysical (the SPR binding assay)-based methods provide useful insights into the overall inhibition capacity of minor cannabinoids against the activity of SARS-CoV-2 3CL M^pro^. Moreover, several minor cannabinoids were identified as promising inhibitors of the SARS-CoV-2 M^pro^, which supports cannabinoids’ potential as lead compounds for the development of preventive and/or therapeutic strategies for COVID-19.

## 4. Materials and Methods

### 4.1. Minor Cannabinoids

Cannabinoids including the major cannabinoid (CBD) and minor cannabinoids, as well as the M^pro^ inhibitors including (M^pro^ 11a, M^pro^ 11b, and M^pro^ 3N) were obtained from Cayman Chemical Company (Ann Arbor, MI, USA).

### 4.2. Enzyme Inhibition Assay

A SARS-CoV-2 3CL main protease (MBP-tagged) assay kit was purchased from BPS Bioscience (San Diego, CA, USA) and used as we reported [31]. In brief, the assay buffer was prepared by adding dithiothreitol, and then the protease enzyme was diluted to a concentration of 3–5 ng/μL. Next, diluted protease enzyme solution was added to a 96-well microplate with the test samples and the positive controls. The assay buffer was added to the wells as blank. The stock solutions of cannabinoids were prepared with the 1× assay buffer. In the first round of screening, cannabinoids were prepared in a stock solution (10 mM in DMSO) and diluted to a final concentration of 10 μM (DMSO concentration was no greater than 0.1%). Three known 3CL M^pro^ inhibitors including M^pro^ 11a, M^pro^ 11b and M^pro^ N3 at a concentration of 10 μM were used as the positive controls. The enzyme was incubated with test compounds at room temperature. The reaction was started by adding the substrate solution to each well followed by recording the optical density of each well using a SpectraMax M2 plate reader at an excitation wavelength of 360 nm and a detection wavelength of 460 nm for 4 hr. Data were expressed as the mean ± standard deviation from three replicates of experiments. GraphPad Prism8 (GraphPad Software, La Jolla, CA, USA) was used to generate the graphics and calculate the IC_50_ values.

### 4.3. Surface Plasmon Resonance

The 3CL M^pro^ protease from coronavirus SARS was purchased from Sigma-Aldrich (St. Louis, MO, USA). The SPR binding measurements were conducted using a Biacore T200 instrument (GE Healthcare, Marlborough, MA, USA) as we reported [31,32]. An SPR sensor chip (PEG coated chip) was activated by a modified amine coupling method. M^pro^ was diluted to 40 μg/mL in an acetate buffer (10 mM; pH 5.0) and coated on the flow cell 2 channel after the injection of the *N*-(3-dimethylaminopropyl)-*N*′-ethylcarbodimide hydrochloride/*N*-hydroxysuccinimide mixture for 30 s. The contact time of M^pro^ on the chip was set to 2100 s and the flow rate was 10 μL/min. A blank immobilization was conducted on Flow cell 1. Three injections of NaOH (50 mM) were performed to remove the unbound M^pro^ protein followed by three cycles of start-up of running buffer injections. The running buffer was phosphate buffered saline (PBS; 10 mM; pH 7.4) containing 3% of DMSO. The stock solutions of cannabinoids were prepared in 2× fold dilutions from 75 to 4.68 μM in running buffer. The sample cycles consisted of steps including sample injection, a buffer flow (i.e., association phase and dissociation phase), and a regeneration pulse (with NaOH; 50 mM) to remove bound analytes on flow cell 2. Solvent correction cycles consisting of eight correction points (2.5% to 3.8% of DMSO) were performed at intervals during the assays to compensate for the differences raised from solvent changing.

### 4.4. Molecular Modeling

The crystal structure of the SARS-CoV M^pro^ protein was retrieved from the protein data bank (PDB ID: 7CAM) with a resolution of 2.85 A°. The Chimera 11.3 software was used to prepare protein for further analysis [33]. The chemical structures of the cannabinoids were obtained from PubChem and the molecular operating environment (MOE) was used to build the 3D structure of ligands. The PDBQT format for protein and ligands was prepared for docking using the Autodock 4.2 algorithm. Pre-calculated grid maps were obtained by adjusting the *x*/*y*/*z* center entries to ensure the ligand was located in the binding pocket as defined by N9 [13]. Next, docking parameters were obtained by Lamarckian genetic algorithm. The binding energy was calculated as estimated by the following formula: Free energy of binding = [(1) + (2) + (3) − (4)], where (1) was the final intermolecular energy, (2) was the final total internal energy, (3) was the torsional free energy, and (4) was the unbound systems. The ligand-target complex with the lowest binding energy was written in a pdb format and the Discovery Studio 4.5 software (Accelrys Inc., San Diego, CA, USA) was used to analyze the interactive residues.

## 5. Conclusions

In summary, the inhibitory effects of a collection of cannabinoids on SARS-CoV-2 3CL M^pro^ were screened by a biochemical assay. Several minor cannabinoids (e.g., THCB and CBGA) showed promising anti-M^pro^ activity. In addition, we observed that decarboxylated cannabinoids, such as CBG and CBD, showed undermined inhibition capacity, as compared to the precursing cannabinoid acids (i.e., CBGA and CBDA, respectively). This SAR was supported by the binding affinities between these cannabinoids and the M^pro^ protein obtained from the SPR assays. Furthermore, the impact of the length of the alkyl side chain of cannabinoids on their anti-M^pro^ activity was explored. Our study is the first to evaluate the anti-M^pro^ activity of minor cannabinoids and their mechanisms of action, which contribute to a better understanding of cannabinoids’ potential roles in the management of COVID-19.

## Figures and Tables

**Table 1:** M^pro^ protein binding parameters (*K*a, *K*d, and *K*_D_) of known SARS-CoV-2 M^pro^ inhibitors obtained from the SPR experiments.

Ligand	*K*a (1/Ms)	*K*d (1/s)	*K*_D_ (M)
M^pro^ 11a	7715	0.1464	1.90 × 10^−5^
M^pro^ 11b	7975	0.05533	6.94 × 10^−6^
M^pro^ N3	6631	0.02898	4.37 × 10^−6^

**Table 2:** M^pro^ protein binding parameters (*K*a, *K*d, and *K*_D_) of CBGA and CBG obtained from the SPR experiments.

Ligand	*K*a (1/Ms)	*K*d (1/s)	*K*_D_ (M)
CBGA	389.8	0.01516	3.89 × 10^−5^
CBG	12160	1.104	9.01 × 10^−5^

**Table 3:** Binding energies between CBGA or CBG and the M^pro^ protein predicted by the molecular docking experiments.

Predicted Binding Energies	CBGA (kcal/mol)	CBG (kcal/mol)
Free binding energy	−4.61	−4.85
Intermolecular energy	−8.48	−8.13
Total internal energy	−2.29	−1.27
Torsional free energy	3.88	3.28

**Table 4:** M^pro^ protein binding parameters (*K*a, *K*d, and *K*_D_) of CBDA and CBD obtained from the SPR experiments.

Ligand	*K*a (1/Ms)	*K*d (1/s)	*K*_D_ (M)
CBDA	12160	1.104	9.08 × 10^−5^
CBD	533.3	0.04173	7.83 × 10^−5^

**Table 5:** Binding energies between CBDA or CBD and the M^pro^ protein predicted by the molecular docking experiments.

Predicted Binding Energies	CBDA (kcal/mol)	CBD (kcal/mol)
Free binding energy	−6.51	−6.85
Intermolecular energy	−9.49	−9.24
Total internal energy	−2.39	−1.29
Torsional free energy	2.98	2.39

## Data Availability

Raw data obtained in this study are available from the corresponding authors upon reasonable request.

## Supplementary Materials

The following supporting information can be downloaded at: https://www.mdpi.com/article/10.3390/molecules27186127/s1. Figure S1: The M^pro^ inhibitory effects of known inhibitors of SARS-CoV-2 M^pro^.

## Sample Availability

Samples of the compounds are not available from the authors.
