# Supplementary material for: Identification of SARS-CoV-2 Main Protease Inhibitors from a Library of Minor Cannabinoids by Biochemical Inhibition Assay and Surface Plasmon Resonance Characterized Binding Affinity
Source: Molecules. 2022 Sep 19;27(18):6127. doi: 10.3390/molecules27186127 (PMC9502466; doi:10.3390/molecules27186127)
Supplement: Supplementary file 1 [file molecules-27-06127-s001.zip › molecules-1905102-supplementary.pdf]

SUPPLEMENTARY MATERIALS

Identification of SARS-CoV-2 Main Protease Inhibitors from a Library of Minor Cannabinoids by Biochemical Inhibition Assay and Surface Plasmon Resonance Characterized Binding Affinity

Chang Liu, Tess Puopolo, Huifang Li, Ang Cai, Navindra P. Seeram*, Hang Ma*

Department of Biomedical and Pharmaceutical Sciences, College of Pharmacy, University of Rhode Island, Kingston, RI 02881, USA

***Correspondence**:

Navindra P. Seeram, Phone: +1(401)-874-9367; Email: nseeram@uri.edu

Hang Ma, Phone: +1(401)-874-7654; Email: hang_ma@uri.edu

Address: 7 Greenhouse Rd, Kingston, RI 02881, USA

Department of Biomedical and Pharmaceutical Sciences, College of Pharmacy, University of Rhode Island

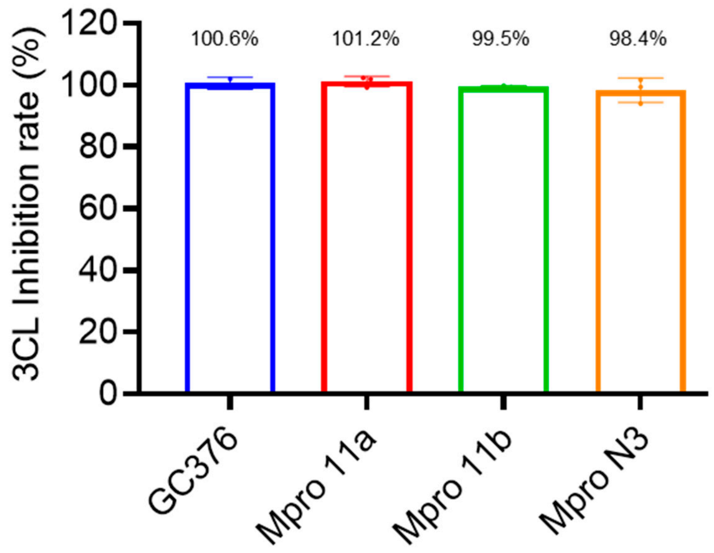

Figure S1. The M^{pro} inhibitory effects of known inhibitors of SARS-CoV-2 M^{pro}. Including GC376, Mpro 11a, Mpro 11b, and Mpro N3 at a concentration of 10 μM.
